# Ultrashort Pulse Laser Fabrication and Evaluation of Innovative Resorbable Barbed Sutures

**DOI:** 10.3390/polym17040544

**Published:** 2025-02-19

**Authors:** Karuna Nambi Gowri, Walid Al Asad, Shubha Majumder, Xin Zhao, Martin William King

**Affiliations:** 1 Department of Textile Engineering, Chemistry and Science, Wilson College of Textiles, North Carolina State University, Raleigh, NC 27606, USA; 2 Department of Mechanical Engineering, Clemson University, Clemson, SC 29634, USA; malasad@g.clemson.edu (W.A.A.); smajumd@g.clemson.edu (S.M.); xzhao5@clemson.edu (X.Z.); 3 College of Textiles, Donghua University, Songjiang District, Shanghai 201620, China

**Keywords:** ultrashort pulse laser, barbed suture, microfabrication, barb geometry, wound closure device, mechanical property, anchoring property

## Abstract

Laser micro-machining is a rapidly growing technique to create, manufacture and fabricate microstructures on different materials ranging from metals and ceramics to polymers. Micro- and nano-machining on different materials has been helpful and useful for various biomedical applications. This study focuses on the micro-machining of innovative barbed sutures using an ultrashort pulse laser, specifically a femtosecond (fs) laser system. Two bioresorbable polymeric materials, namely, catgut and poly (4-hydroxybutyrate) (P4HB), were studied and micro-machined using the femtosecond (fs) laser system. The optimized laser parameter was used to fabricate two different barb geometries, namely, straight and curved barbs. The mechanical properties were evaluated via tensile testing, and the anchoring performance was studied by means of a suture–tissue pull-out protocol using porcine dermis tissue which was harvested from the medial dorsal site. Along with the evaluation of the mechanical and anchoring properties, the thermal characteristics and degradation profiles were assessed and compared against mechanically cut barbed sutures using a flat blade. The mechanical properties of laser-fabricated barbed sutures were significantly improved when compared to the mechanical properties of the traditionally/mechanically cut barbed sutures, while there was not any significant difference in the anchoring properties of the barbed sutures fabricated through either of the fabrication techniques. Based on the differential scanning calorimetry (DSC) results for thermal transitions, there was no major impact on the inherent material properties due to the laser treatment. This was also observed in the degradation results, where both the mechanically cut and laser-fabricated barbed sutures exhibited similar profiles throughout the evaluation time period. It was concluded that switching the fabrication technique from mechanical cutting to laser fabrication would be beneficial in producing a more reproducible and consistent barb geometry with more precision and accuracy.

## 1. Introduction

A laser produces a high-energy narrow beam of light that is useful in many technologies and instruments. The letters in the word laser stand for “Light Amplification by Stimulated Emission of Radiation”. This means that a laser is a device that emits light from a coherent source, and the light beam is focused onto a very limited area. This makes the use of a laser ideally suited for precise manufacturing techniques and lithographic applications. Lasers are currently being used in a wide variety of industries, from semiconductors to metals and from communication systems to medical procedures. In recent times, the use of lasers in surgical procedures and other biomedical applications has increased tremendously, since lasers can be used to penetrate through a surface with precise focus and reliable accuracy without disturbing the surroundings. In fact, lasers have become one of the most important techniques for material processing, since a laser can be used to drill an extremely small and deep hole and for the application and attachment of thin films and nanoparticles onto material surfaces in a clearly defined and precisely aligned fashion [1].

**Figure 1:**
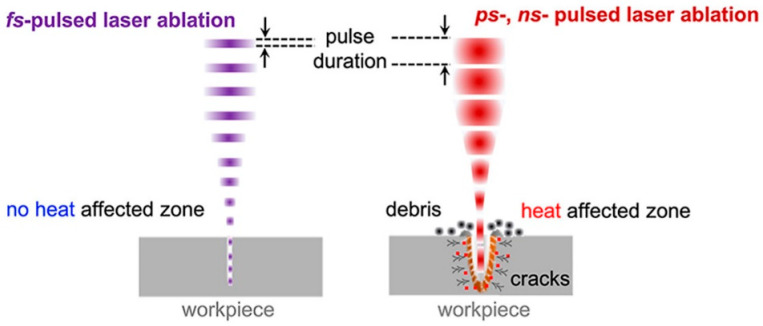
Illustration distinguishing between pulse durations in terms of damage to neighboring material. (**left**) a femtosecond pulsed laser system. (**right**) a picosecond (*ps*) or nanosecond (*ns*) pulsed laser system [4].

Laser micro-machining is a well-sought-after industrial fabrication technique that is used to produce minute and intricate parts in the automotive, aerospace, industrial, electrical and electronics industries. The type of laser suitable and ideal for micro-machining is the class of ultrashort pulsed lasers (USPLs). This class of lasers includes femtosecond lasers (fs), which are preferred for micro-machining since these lasers have minimal or negligible heat-affected zones (HAZs) on the surrounding substrate. Pulsed laser micro-machining is an effective and essential technique for fabricating devices like stents, catheters, micro-electromechanical systems (MEMS) and microfluidic devices, all of which require the precise removal of localized material. The micro-machining of microstructured medical devices has evolved and become possible using different types of pulsed lasers [2,3]. The ability of ultrashort lasers to machine or fabricate sub-micron features on a wide range of materials has a number of technological and medical applications. One of the most promising machining methods is based on the pulsed laser ablation principle, and the femtosecond (fs) laser is widely preferred for micro-machining ceramics, metals and polymeric films as it avoids causing thermal damage because of the negligible heat-affected zone in the surrounding bulk material (Figure 1).

The micro-machining and micro-texturing of materials are routinely performed using ultrashort pulsed lasers, particularly picosecond and femtosecond lasers. Such micro-machining is able to undertake the non-thermal removal of material and high-precision ablation with reduced thermal damage to the surrounding material [5]. Micro-machining is now a part of the production process on the industrial floor, since the fabrication of microstructures and nanostructures was more efficient and more precise when performed using an ultrashort pulsed laser. Due to the uniqueness of this class of laser, it is possible for the ablation process to undertake cold ablation and/or to generate a very precise heat load for thermally sensitive materials [6]. Short-pulsed lasers have major advantages over long pulse lasers, such as their short interaction time, minimal heating zone and precise dimensional structures [1]. Ultra-fast lasers offer high laser intensity and a precise laser-induced breakdown threshold with reduced laser fluence. The extremely short pulse width makes it easy to achieve very high peak laser intensities with a very low pulse energy. Ultrashort pulsed lasers can be used to machine substrates at the wavelength where substrates become transparent in nature [7,8].

It was previously determined that femtosecond laser systems have a very low laser fluence threshold, making them suitable for certain machining situations where low fluences are desired to minimize collateral damage. For example, in medical surgery, where tissue damage outside the region of interest needs to be at a minimum, it has previously been demonstrated that femtosecond laser systems can be utilized to produce microstructures with outstanding surface qualities in terms of the achievable minimum diameter, maximum workpiece length and short processing time. In fact, it was concluded that femtosecond laser-based machining offers increased fabrication speed and reduced operating time compared to other alternative fabrication techniques used for producing microstructures [9,10]. Due to all these advantages over other fabrication techniques, the femtosecond laser is an attractive tool to create structures on biological tissues without damaging the bulk tissue [11]; to create patterns on biopolymeric surfaces to alter the surface characteristics [12]; to deposit labile biomaterials on different substrates without significant damage [13]; and to enable three-dimensional direct cell patterning and microstructuring on polymers and hydrogels for effective cell proliferation and migration into the cellular architecture using an appropriate material removal technique [14,15,16].

Ultrashort pulse laser microfabrication on polymeric materials is an interesting technique to fabricate micro- and nanostructures without greatly affecting the entire substrate or its inherent properties. This fabrication method is gaining interest among researchers working in the biomedical, automotive and aerospace industries and other areas where minute fabrication is required without damaging the substrate in its entirety. The present study focuses on the ultrashort femtosecond pulsed laser fabrication technique for barbing sutures from bioresorbable polymers such as catgut [17,18] and poly(4-hydroxybutyrate) (P4HB) suture materials. The femtosecond IR laser system was optimized and used to fabricate barbed sutures with two different barb geometries, which were then evaluated for their tensile mechanical properties and anchoring performance by an in vitro suture–tissue pull-out test protocol [19]. It was mentioned in a previous patent published by Harry J. Buncke [20] that barbed sutures could be fabricated using a laser system, but the fabrication technique using a laser system was never implemented or practically evaluated for the manufacturing of barbed sutures. The novelty of this research study is that it is the first to practically fabricate barbed sutures using a laser system and to evaluate their tensile and anchoring performance. In addition, this is the first study which manipulates the laser parameters to fabricate barbed sutures with varying barb geometries and then evaluates their tensile and anchoring properties. And the main purpose of this research is to understand and evaluate the practical feasibility of fabricating barbed sutures using a laser fabrication technique.

## 2. Materials and Methods

### 2.1. Materials Used

The materials used in this study were bioresorbable polymeric monofilaments made from poly(4-hydroxybutyrate) (P4HB) and catgut filaments. The catgut monofilaments, size 0 (ϕ: 0.400 mm–0.499 mm) and size 2-0 (ϕ: 0.339 mm–0.400 mm), were supplied by Ethicon Inc., Somerville, NJ, USA. Catgut or preserved collagen surgical sutures have been used for wound closure since the origins of medicine back in 300 BC [17,18,21]. On the other hand, size 2-0 (ϕ: 0.300 mm–0.339 mm) P4HB monofilaments (B. Braun Medical Inc., Barcelona, Spain) are a relatively new material that was approved by the US Food and Drug Administration (FDA) in the year 2007 for use as a resorbable suture material [22,23].

### 2.2. Femtosecond Laser Fabrication

#### 2.2.1. Femtosecond Laser Schematic

**Figure 2:**
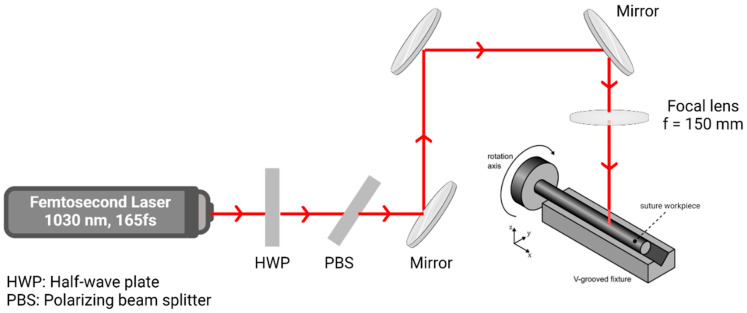
Schematic illustration of the femtosecond infrared laser used to fabricate barbed sutures.

The femtosecond laser (Pharos: by Light Conversion, Vilnius, Lithuania) suitable for use in the experimental study to fabricate barbs on monofilament sutures was identified to be available at the Laser-Based Manufacturing and Materials Processing Laboratory, at Clemson University. This laser is an infrared (IR) femtosecond laser, with a wavelength of 1030 nm and a pulse duration of 165 fs. This is referred to as a full width at half maximum (FWHM)-intensity laser. A schematic diagram of the fs laser available at Clemson University is shown in Figure 2. The laser scanner (Scanlab laserDESK, München, Germany) was linked to the femtosecond laser using a half-wave plate, a polarizing beam splitter and a series of mirrors and a lens which focused the beam on the monofilament held at the bottom of a V-shaped grooved fixture.

#### 2.2.2. Optimum Laser Parameters for Barbed Suture Fabrication

For the purpose of fabricating barbed sutures, four laser parameters were carefully optimized to achieve superior machining quality and operative precision. These parameters include the laser fluence, the repetition rate, the overlapping ratio and the number of scanning passes. The laser fluence refers to the amount of energy delivered per unit area, which is crucial for precise material removal. The repetition rate is the frequency at which laser pulses are emitted, and it is directly correlated with the processing time. The overlapping ratio indicates the extent of coverage between two consecutive laser pulses, which impacts the uniformity of the surface. And lastly, “scanning passes” describes the number of times a laser beam traverses over a target area, which affects the depth and the quality of the cut. The optimized laser parameters used in the fabrication process are listed in Table 1.

### 2.3. Barb Geometry

**Figure 3:**
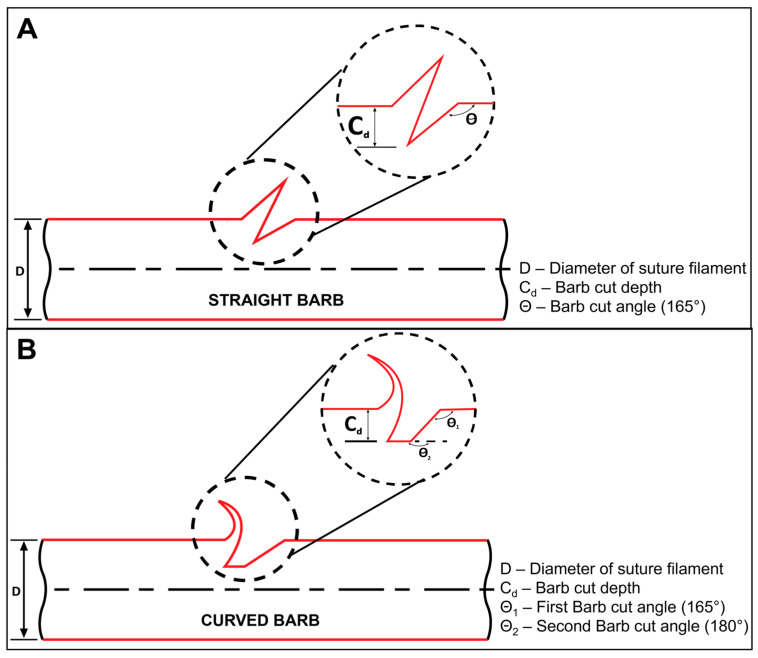
Illustration representing the important parameters of a single barb: (**A**) straight barb and (**B**) curved barb [24].

In this study, barbed sutures were fabricated with both straight and curved barb geometries using a mechanical barbing instrument [24] and the femtosecond (fs) laser system shown above in Figure 2. When barbs were cut mechanically, the frequency, angle and depth of the barbs (Figure 3) were fixed and determined by the blade assembly, which consisted of 9 blades mounted equidistant from each other within a 4 cm distance on a base plate. For a bidirectional suture, the barbed sections were 7 cm in length facing opposite directions at both ends with a 2 cm gap in the middle. The straight barbs were cut with the following parameters: the cut depth was fixed at 20% of the suture diameter and the cut angle was fixed at 165° ± 5°.

Curved barbs were fabricated using the same initial barbing instrument and blade assembly with the same 20% cut depth and 165° cut angle. After straight barbs were produced, a second linear cut was made at 180°, i.e., parallel to the suture axis, so as to increase the barb length and thereby develop a curved barb shape. In Figure 3, we can observe the difference in the cut angle used in order to fabricate the straight and curved barb geometries. After barbing, the sutures were swaged with size 22 taper-pointed needles for size 2-0 sutures and size 24 taper-pointed needles for size 0 sutures. These needle sizes are frequently used for wound closure.

### 2.4. Optical Microscopy

After fabrication, the barbed sutures were viewed at 5× and 10× magnification under a high-resolution EVOS FL Auto2 cell-imaging microscope (Thermo Fisher Scientific, Waltham, MA, USA). This provided optical microscopic images of the barb shape and geometry for the sutures that were fabricated using the mechanical barbing instrument compared to those fabricated on the femtosecond laser stage.

### 2.5. Tensile and Anchoring Property Evaluation

#### 2.5.1. Suture Tensile Test

The tensile properties of the laser-fabricated barbed and non-barbed sutures were measured and compared against commercial barbed polypropylene sutures on an MTS Criterion Model 43 tester (MTS Systems, Eden Prairie, MN, USA) with a 500 N load cell following the ASTM D2256/D2256M—2021 “Standard Test Method for Tensile Properties of Yarns by the Single-Strand Method” [25]. The suture specimens were mounted between capstan clamps at a pressure of 60 psi, a gauge length of 5 inches (12.7 cm) and a crosshead speed of 150 mm/min. The mechanical properties of ultimate tensile force and elongation at break were measured and recorded. In addition, the Young’s modulus, which measures the initial resistance to stretching, was determined from the slope of the recorded stress–strain curve, and the work to rupture or toughness was calculated from the area under the load–displacement curve. In this study, suture stiffness refers to the suture’s resistance to deformation when a tensile load is applied, and toughness measures how much energy the suture absorbs prior to breakage at the failure point.

#### 2.5.2. Suture–Tissue Pull-Out Test (Anchoring Performance Evaluation)

In order to mimic the in vivo anchoring performance of barbed sutures within their surrounding tissue, porcine dermis was used in an in vitro protocol to evaluate the suture–tissue pull-out strength. The samples of dermis tissue were kept in phosphate-buffered solution (PBS) as soon as they were harvested from the mid-dorsal region of the pig at the College of Veterinary Medicine, North Carolina State University. A number of 3/8 taper point needles were swaged for both bidirectional barbed and non-barbed sutures. The suture bite distance was calculated to be 0.804 mm for a size 2-0 suture and 0.941 mm for a size 0 suture. The anchoring properties of the mechanically barbed and non-barbed sutures were measured and compared against commercially available barbed sutures on an MTS Q-test/5 mechanical tester (MTS Systems, Eden Prairie, MN) with a modification to the flat-faced clamps on the test instrument. Anchoring performance was measured in terms of the maximum pull-out load and elongation using the ASTM D3822/D3822M—2020 “Standard Test Method for Tensile Properties of Single Textile Fibers” [26]. One suture end was attached to the top clamp, while the porcine dermis was held by the bottom clamp, with sandpaper lining the clamp surfaces so as to avoid the slippage of the dermis at a pressure of 50 psi. The standard uniaxial tensile test was performed with a gauge length of 2 inches (7.62 cm) and a crosshead speed of 150 mm/min. The maximum pull-out force at failure was considered a direct measurement of suture anchoring performance with the surrounding tissue.

### 2.6. In Vitro Hydrolytic Degradation Evaluation

The resistance to hydrolytic degradation was evaluated for the catgut and poly(4-hydroxy-butyrate) (P4HB) sutures with the objective of determining whether or not the barbs fabricated mechanically degraded earlier than the non-barbed suture monofilaments. The suture materials of catgut and P4HB are known to experience degradation through a hydrolytic mechanism. An in vitro hydrolytic degradation protocol was performed in order to determine the degradation profiles of both P4HB and catgut monofilaments.

The non-barbed and barbed suture monofilaments were incubated at 37 °C in 25 mL of phosphate-buffered solution (pH = 7.4) for up to six weeks for the P4HB sutures and for up to 10 days for the catgut sutures. The degradation solution was renewed after 0, 1, 2, 3, 4, 5 and 6 weeks for the P4HB samples and after 0, 2, 4, 6, 8 and 10 days for the catgut sutures. At each time point, the ultimate tensile force (N) of the fabricated barbed sutures was measured and compared against their respective non-barbed counterparts.

### 2.7. Differential Scanning Calorimetry (DSC) Analysis

The thermal behavior of the monofilament sutures was characterized by differential scanning calorimetry (DSC) using a Discovery DSC 250 instrument (TA Instruments, New Castle, DE, USA), an analytical instrument that is used to measure “heat flow”, or the thermal energy that is absorbed or released by a substrate as a function of temperature or time. The TA Instruments Discovery DSC 250 is a DSC instrument with a refrigerated cooling system (RCS) that provides cooling to −90 °C and is a heat flux instrument with an autosampler. This instrument is typically used to analyze the thermal transitions that occur within polymeric materials, namely, the glass transition temperature (T_g_), the melting temperature (T_m_), the crystallization temperature (T_c_) in the case of thermoplastic polymeric filaments and the degradation temperature (T_d_) in the case of thermoset polymeric filaments. The suture monofilaments, both barbed and non-barbed, were cut into short lengths which weighed between 5 and 10 mg. The heating and cooling rates were maintained at 10 °C/min, and all the samples were heated up to 200 °C–225 °C, following the ASTM D3418 “Standard Test Method for Transition Temperatures and Enthalpies of Fusion and Crystallization of Polymers by Differential Scanning Calorimetry” [27]. Through the use of this standard test method, the glass transition temperature (T_g_), melting temperature (T_m_) and degradation/decomposition temperature (T_d_) were measured and reported.

### 2.8. Statistical Analysis

Barb geometries, mechanical properties and anchoring performance results were compared between the barbed and non-barbed suture controls for each of the respective materials: P4HB and catgut. The data were also compared against the commercially available polypropylene Quill^TM^ barbed 2-0 sutures. Each group had four specimens on which statistical analysis needed to be performed. The statistical analysis was performed using a one-way ANOVA with Tukey’s adjustments with a *p*-level of 0.05 using Origin software 2023 (Academic) (Origin Lab, Northampton, MA, USA). The average data are reported as mean ± standard deviation.

## 3. Results

### 3.1. Optimal Approach for Laser Fabrication of Barbed Sutures

**Figure 4:**
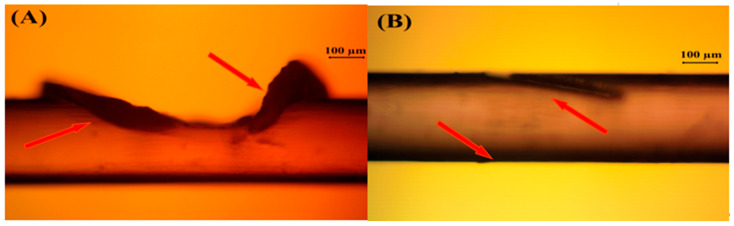
Laser-treated P4HB 2-0 sutures with the following ablation parameters: (**A**) laser fluence of 29.74 J/cm^2^, laser pulse energy of 15 µJ, overlapping ratio of 50% and 50 scanning passes; (**B**) laser fluence of 3.3 J/cm^2^, laser pulse energy of 15 µJ, overlapping ratio of 50% and 140 scanning passes (note: red arrows represent the barbs cut on P4HB sutures).

The laser fabrication of barbed sutures was performed using the optimized set of parameters listed in Table 1 for both P4HB and catgut suture monofilaments. While optimizing the parameters, we observed that in order to produce sharper, finer and better-quality barbs, a lower laser power was needed along with an increasing number of scans. Through this means, higher-quality barbs were achieved without disrupting the suture material and its inherent properties. This can be readily seen in Figure 4. The barbs shown in Figure 4 were machined using a repetition rate of 10 kHz and 70% overlapping ratio, but with different laser fluences (29.74 J/cm^2^, 3.30 J/cm^2^) and a different number of scans (50, 140).

### 3.2. Optical Microscopy—Variation in Barb Geometry

Different barb geometries were created depending on the material and fabrication technique used to produce the barbs. In the case of the mechanical cutting technique, the barbed sutures with straight barbs were fabricated using the stationary straight blade assembly consisting of nine blades mounted at a fixed angle. This approach generates barbs at a specific cut angle and barb depth controlled by the extent to which the monofilament protrudes above the groove of the barbing assembly (Figure 3). In the case of the laser fabrication technique, the laser pathway was aligned to form an array of barbs similar to those created by the stationary blades [24]. Curved barbs were fabricated mechanically by using two cut angles as shown in Figure 3 [24], and for laser fabrication, the laser pathway was modified to follow the same two cut angles.

**Figure 5:**
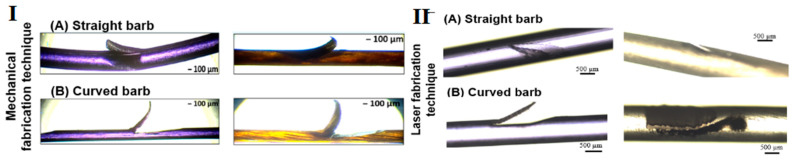
P4HB and catgut barbed sutures fabricated using (**I**) a mechanical cutting technique for (**A**) straight barbs and (**B**) curved barbs, and using (**II**) a laser fabrication technique for (**A**) straight barbs and (**B**) curved barbs.

Figure 5 shows images of straight and curved barbs fabricated on both P4HB (violet) and catgut (yellow) sutures using both the mechanical cutting technique and the laser technique. The series of images in Figure 5 compares the structure of the barbs fabricated by mechanical cutting and by the laser fabrication technique. The mechanically cut barbs, both straight and curved, demonstrate inconsistent barb cut depth, while laser-cut barbs have consistent barb cut depth.

### 3.3. Tensile and Anchoring Properties

#### 3.3.1. Suture Tensile Properties

**Figure 6:**
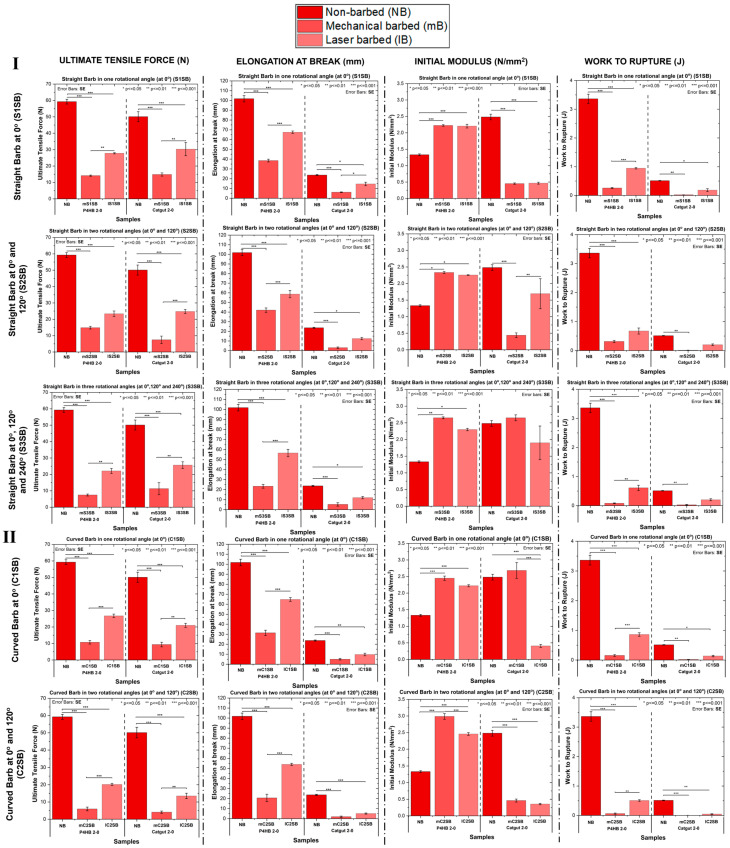
Comparison of ultimate tensile force (N), elongation at break (mm), initial modulus (N/mm^2^) and work to rupture (J) of samples with different barb geometries and rotational angles fabricated by both mechanical cutting and laser erosion techniques. (**I**): Straight barbs fabricated using one, two, and three rotational angles. (**II**): Curved barbs fabricated using one and two rotational angles.

The tensile results of the mechanically fabricated and laser-fabricated barbed sutures were compared with those of non-barbed sutures and are shown in Figure 6. The results for the ultimate tensile force (N), elongation at break (mm), initial modulus (N/mm^2^) and work to rupture (J) were compared based on the barb geometry and number of rotational angles used. Figure 6 shows that the mechanical properties of the laser-fabricated barbed sutures were significantly more enhanced than those of the mechanically cut barbed sutures. The ultimate tensile force (N) (Figure 6I,II) was significantly reduced after barbing irrespective of the polymer material and the fabrication technique. In the plots, it can be seen that there was a 75% loss for the straight and curved barbed sutures (*p* < 0.001). All the barbed sutures were compared to their respective non-barbed controls. The elongation at break (%) and the toughness or work to rupture (J) were significantly reduced after barbing irrespective of the polymer material, and the percent change followed a trend similar to the decrease observed in the ultimate tensile force results. The stiffness or initial tensile modulus (N/mm^2^) (Figure 6I,II) of the sutures showed an increase of about 18% (*p* < 0.001) after barbing compared to the non-barbed controls.

#### 3.3.2. Suture–Tissue Pull-Out Test (Anchoring Performance Evaluation)

**Figure 7:**
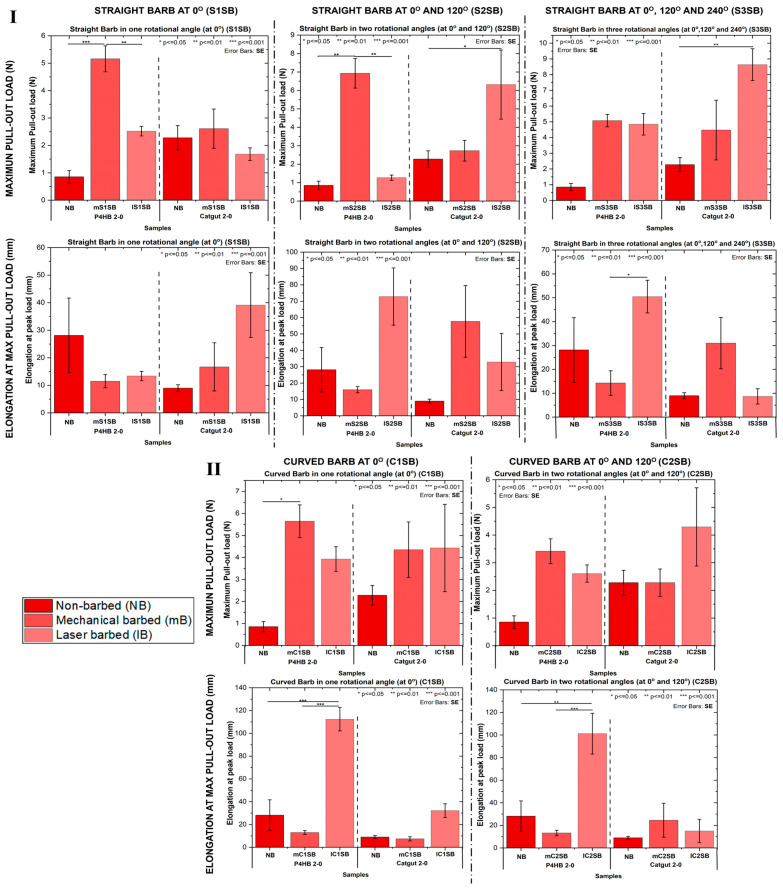
A comparison of anchoring properties, namely, the maximum pull-out load (N) and the elongation at break at the maximum pull-out load (mm), of samples with different barb geometries and rotational angles fabricated by both mechanical cutting and laser erosion techniques. (**I**): Straight barbs fabricated using one, two, and three rotational angles. (**II**): Curved barbs fabricated using one and two rotational angles.

Through the application of the suture–tissue pull-out test, the suture anchoring performance in porcine dermal tissue was determined experimentally. The maximum pull-out load (N) and the elongation at break at the maximum pull-out load (mm) of the mechanically fabricated and laser-fabricated barbed sutures were compared based on the barb geometry and number of rotational angles used as shown in Figure 7. Invariably, the barbed sutures, with their higher maximum pull-out load (Figure 7I,II), showed superior anchoring performance because the barbs engaged with the surrounding tissue. In the plots shown in Figure 7, it can be observed that the fabricated straight barbed sutures showed around 40% (*p* < 0.001) higher resistance in tissues compared to their non-barbed counterparts which slipped through the suture incision point. An increase in the maximum pull-out load (N) of about 42% (*p* < 0.001) was observed for the fabricated curved barbed sutures as well. This trend of increase was also seen in the elongation at break at the maximum pull-out load (mm) in the case of both straight and curved barbed sutures fabricated through both the fabrication techniques.

### 3.4. In Vitro Hydrolytic Degradation Evaluation

**Figure 8:**
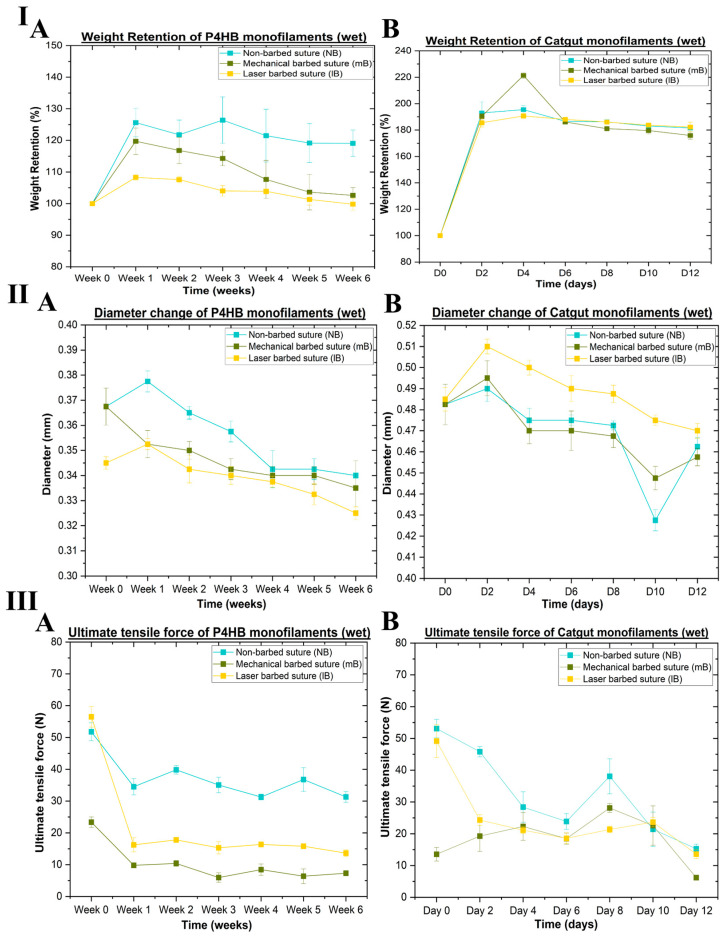
Results of hydrolytic degradation evaluation under wet conditions. (**I**) Weight retention (%) for (**A**) P4HB and (**B**) catgut sutures. (**II**) Diameter change (mm) for (**A**) P4HB and (**B**) catgut sutures. (**III**) Ultimate tensile force (N) for (**A**) P4HB and (**B**) catgut sutures.

This in vitro evaluation was performed to determine the degradation profiles of the mechanically fabricated and laser-fabricated barbed sutures compared with the non-barbed sutures. At each time point, the P4HB and catgut barbed sutures were weighed, and their monofilament diameter and tensile properties were determined under wet conditions. This facilitated the calculation of weight retention, changes in diameter and ultimate tensile force for the P4HB and catgut sutures, which are plotted as a function of the hydrolysis time in Figure 8. Figure 8I(A,B) illustrates the changes in weight retention (%), Figure 8II(A,B) show the diameter changes, and Figure 8III(A,B) represent the variations in the ultimate tensile force (N) for both suture materials over time.

### 3.5. Differential Scanning Calorimetry (DSC) Analysis

**Figure 9:**
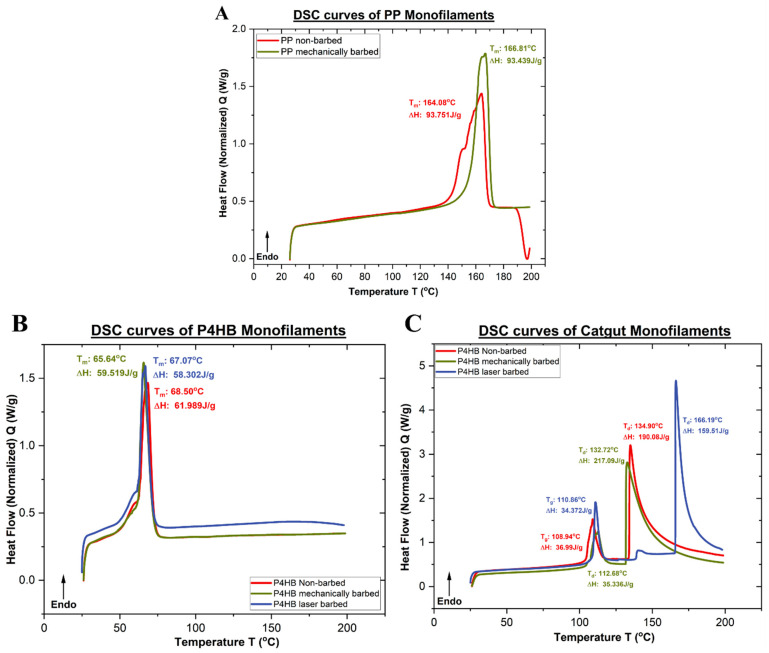
DSC thermograms for (**A**) polypropylene (PP), (**B**) poly(4-hydroxybutyrate) (P4HB) and (**C**) catgut sutures before and after barbing.

The thermal behavior of the polymeric filaments was determined by DSC analysis in order to understand if there was a change in the thermal transition temperatures caused by barbing. Measurements were performed on polypropylene (PP), P4HB and catgut sutures before and after mechanical and laser barbing using the standard test method ASTM D3418. Figure 9A–C represent the thermograms obtained for the PP, P4HB and catgut sutures, respectively.

## 4. Discussion

Femtosecond (fs) laser fabrication is a rapidly advancing technique to micro-machine materials ranging from metals and ceramics to polymers. In this study, the laser fabrication of barbed sutures was performed in order to reduce the barbed suture manufacturing time and to increase the efficiency of producing barbed sutures. This is the first study to conduct and evaluate the application of a femtosecond (fs) laser microfabrication technique to create barbed sutures without disrupting the inherent material characteristics of the polymeric suture material. It was observed that when a high laser fluence together with fewer scanning passes was employed, the increased thermal energy caused damage to the surrounding areas, which resulted in a barb that was barely recognizable (Figure 4A). On the contrary, when a lower laser fluence and more scanning passes were used, it was possible to generate high-quality barbs (Figure 4B). Consequently, it can be assumed that the optimal approach to producing sharper, finer and superior-quality barbs is to use a lower laser fluence with more scanning passes. While it is evident that the use of a high laser fluence and fewer scanning passes can provide rapid ablation and reduced processing time, it was also observed that these conditions cause a significant deterioration in the quality of the barbs.

The structure and geometry of the barbs fabricated through both techniques were evaluated under an optical microscope. In the optical micrographs illustrated in Figure 5I,II, we observed that the barbs fabricated by mechanical cutting were more open than the laser-fabricated samples. At the same time, the barbs fabricated through laser ablation were more precise in terms of their cut depth and cut angle. In fact, the laser fabrication technique was able to prepare barbs with a more consistent cut depth and cut angle, so it was possible to reproduce barbed sutures with superior accuracy, precision and efficiency. In Figure 5I,II, it can also be observed that despite having the same dimensions for barb cut angle and cut depth, the laser-cut barbs were shallower and closer to the monofilaments, unlike the mechanically cut barbs that were more open, deeper and stood out away from the filament when observed under optical microscopy.

The tensile properties and anchoring characteristics of the three groups with a straight barb geometry and the two groups with a curved barb geometry were compared in order to determine whether changing the fabrication technique would impact these characteristics. In comparing Figure 6I,II, it can be seen that there was a significant difference in the ultimate tensile force (N), elongation at break (mm) and work to rupture (J) between the mechanically fabricated and laser-fabricated barbed sutures. On the other hand, there was no significant difference between the initial modulus (N/mm^2^) for the mechanically barbed and laser-barbed sutures with straight barbs, whereas a significant difference in the initial modulus was observed for the sutures with curved barbs regardless of the fabrication technique (Figure 6I,II). The laser-fabricated barbed sutures had superior tensile properties compared to the mechanically cut sutures in the case of curved barbs that were fabricated with different numbers of rotational angles.

When comparing the anchoring performance of the size 2-0 P4HB and catgut monofilament barbed sutures, both the maximum pull-out force (N) and the elongation at the maximum pull-out force (mm) were higher for the mechanically barbed sutures, as shown in Figure 7. This was particularly true in the case of fabricating curved barbs both at one rotational angle of 0° and at two rotational angles of 0° and 120°.

Since the use of a laser involves applying thermal treatment, it was essential to study the influence of the laser on the polymeric materials and to evaluate whether switching to a laser barbing technique would compromise the inherent structure and thermal properties of the material. The thermal behavior of the suture material was monitored by differential scanning calorimetry (DSC) before and after the laser barbing procedure. The peaks present in the DSC thermograms show the various exothermic and endothermic transitions that occur when the samples are subjected to thermal treatments. The melting point, glass transition point and polymeric degradation occur as endothermic transitions, while crystallization results in an exothermic regime. Figure 9A represents the DSC analysis performed on the polypropylene (PP) sample before and after barbing with a mechanical blade as well as with laser ablation, while Figure 9B represents the DSC analysis performed on the P4HB samples, and Figure 9C shows the results of the DSC analysis performed on the catgut samples before and after barbing. As observed in Figure 9, the thermal transition peaks became sharper for the barbed suture samples. The reason behind this phenomenon is the fact that the outer material is removed from the main monofilament when the barb projections are cut using the blade assembly. In the case of laser-fabricated P4HB sutures, the thermal transition peaks did not shift, confirming that the laser-treated P4HB sutures did not change their inherent material characteristics. However, for the laser-fabricated catgut sutures, there was a shift in the thermal transition peaks which was not observed with the mechanically fabricated barbed sutures. This shift in the peaks can be explained by the fact that the catgut sutures, being thermoset in nature, were heat-set at a higher temperature due to the laser treatment, even though the femtosecond lasers have negligible heat-affected zones.

An in vitro hydrolytic degradation evaluation was performed to assess the degradation profile of the suture material and to determine whether creating barbs on the periphery of the monofilaments would impact their degradation profile. In Figure 8I,II, it can be seen that there was an increase in both the suture weight and diameter for both the barbed and non-barbed samples during the first couple of time points when immersed in phosphate-buffered solution (PBS). This increase in both weight and diameter is attributed to the swelling of the filaments when first introduced into the PBS. Figure 8III represents the tensile results for the P4HB and catgut samples under wet conditions before and after mechanically barbing and laser barbing the sutures. Both strategies generated a similar trend in terms of the observed ultimate tensile force, indicating that switching the barbed suture fabrication technique to a laser approach did not affect the degradation profiles of either the P4HB or catgut sutures.

## 5. Conclusions

In conclusion, this study compared the structure and properties of barbed sutures fabricated both mechanically and using a laser ablation technique. A comparison of the tensile and anchoring properties of the barbed sutures fabricated by both techniques showed that there is still room for improvement in the case of the laser fabrication approach in order to achieve a similar anchoring performance to that obtained for the mechanically cut barbed sutures. Through the hydrolytic degradation study, it was concluded that the barbs remained intact and attached to the body of the suture. They did not degrade faster and separate from the suture itself. This study also demonstrated that the laser fabrication technique could be used as an attractive alternate strategy to the current mechanical cutting approach using fixed blades, and produce more consistent, highly precise barbs on the periphery of the suture. It was also observed that the femtosecond infrared laser system did not cause major thermal deterioration of the suture material and was an attractive approach for fabricating barbed sutures with minimal thermal damage to the surrounding bulk material.

## Figures and Tables

**Table 1:** Optimized laser parameters for the fabrication of barbed sutures.

Materials (Sutures)	Barb Geometry	Laser Fluence (J/cm^2^)	Repetition Rate (kHz)	Overlapping Ratio (%)	Scanning Passes
P4HB (size 2-0)	Straight	3.30	10	50	140
	Curved	6.60	10	50	300
Catgut (size 2-0)	Straight	9.90	10	50	100
	Curved	11.00	10	50	160
Catgut (size 0)	Straight	9.90	10	50	120
	Curved	11.00	10	50	350

## Data Availability

The original contributions presented in this study are included in the article. Further inquiries can be directed to the corresponding authors.
